# A magic pill? A qualitative analysis of patients’ views on the role of antidepressant therapy in inflammatory bowel disease (IBD)

**DOI:** 10.1186/1471-230X-12-93

**Published:** 2012-07-20

**Authors:** Antonina A Mikocka-Walus, Andrea L Gordon, Benjamin J Stewart, Jane M Andrews

**Affiliations:** 1 School of Nursing and Midwifery, Sansom Institute for Health Research, University of South Australia, Adelaide, Australia; 2 School of Psychology, University of Adelaide, Adelaide, Australia; 3 IBD Service, Department of Gastroenterology and Hepatology, Royal Adelaide Hospital, Adelaide, Australia; 4 School of Medicine, University of Adelaide, Adelaide, SA, Australia

**Keywords:** Antidepressants, Disease activity, Inflammatory bowel disease, Mental health, Patients’ views

## Abstract

**Background:**

Studies with healthy volunteers have demonstrated that antidepressants can improve immunoregulatory activity and thus they may have a potential to positively impact the disease course in inflammatory bowel disease (IBD), a chronic and incurable condition. However, patients’ views on the role of antidepressants in the management of their IBD are unknown. Thus, this study aimed to explore patients’ experiences and opinions regarding the effect of antidepressants on IBD course before possibly undertaking future treatment trials with antidepressants.

**Methods:**

Semi-structured in-depth interviews with open-ended questions were conducted with a randomly selected sample of IBD patients recruited at the Australian public hospital IBD clinic and currently receiving antidepressants. A qualitative content analysis was undertaken to summarise patients’ responses. A Visual Analogue Scale was used to provide a quantitative assessment of patients’ experiences with antidepressants.

**Results:**

Overall, 15 IBD sufferers currently on antidepressants (nine females, six males) were interviewed. All 15 reported a positive response to antidepressants reporting they improved their quality of life, with minimal side-effects. Five patients (33.3%) felt the antidepressant had specifically improved their IBD course. Three patients noted how they believed the reduction in feelings of stress mediated the positive influence of the antidepressant on IBD course. Ten patients (66.7%) felt the antidepressants had not specifically influenced their IBD. Nine patients (60.0%) had a generally positive attitude towards antidepressants, four patients (26.7%) were ambivalent, and two patients (13.3%) held a negative view towards antidepressants. Twelve patients (80.0%) stated that they would be willing to participate in clinical trials.

**Conclusions:**

Antidepressants seem to be well tolerated by IBD patients. One third of patients reported an observable improvement of their IBD under the influence of this treatment. The positive attitude towards antidepressants in these participants may make the conduct of clinical trials to further assess for any specific role on IBD course feasible. However, due to a small sample size, a qualitative nature of this study and in light of the results of studies on other populations indicating reluctance to taking antidepressants at least in some patients, these results should be interpreted with caution until confirmed in quantitative studies.

## Background

Inflammatory bowel disease (IBD) is a group of chronic, relapsing inflammatory disorders of the gut. The aetiology of IBD is unknown, with genetic, immune and environmental factors considered as important contributors to its occurrence. Psychological status has been found to influence the timing of disease relapse [1,2]. Studies show a 30% rate of depression during remission [1], with 80% and 55% of patients reporting anxiety and depression, respectively, during relapse [3]. While studies into psychological therapies [4] have recently been conducted, antidepressants have not been widely studied in the context of IBD. However, studies with healthy volunteers have demonstrated that antidepressants can improve immunoregulatory activity [5] and thus there is a potential for antidepressants to not only help with psychological difficulties but also positively impact the disease course.

Two systematic reviews analysing human and animal studies have been conducted exploring the role of antidepressants in IBD, [6,7]. In humans, it was observed that although antidepressants seem to improve both mental and somatic status of IBD patients, the low quality of available research provides significant barriers to making a definitive statement on their efficacy or lack thereof [7]. Animal models however, found a positive impact of antidepressants (i.e. desipramine and fluoxetine) on inflammation in models of IBD [6]. When doctors’ perspectives on antidepressants in IBD were examined, it was reported that gastroenterologists commonly treat IBD patients with antidepressants for pain, anxiety and/or depression, and insomnia [8]. Gastroenterologists reported that antidepressants were successful in reducing pain, gut irritability, and urgency of defecation. In the most recent retrospective case-note audit, of 287 patients, 83 (28.9%) were found to have used an antidepressant at some time in their life [9]. However, the design of the study does not allow one to make a firm statement on whether antidepressants improved IBD course. The most recent study in this area [10] examining the disease course a year before and a year after the commencement of antidepressants showed that patients reported fewer relapses and steroid treatment in the year after starting an antidepressant than in the year before, which was not observed in the control group. Whilst this report of decreased symptoms may simply reflect the report of fewer functional gastrointestinal symptoms when patients are in better psychological health [11,12], it may also indicate an inflammation-specific benefit from antidepressants. It is thus now clear that randomised controlled trials are justified and needed to provide the definitive answer on the efficacy of antidepressants in IBD.

However, prior to embarking on a clinical trial in this field we felt it prudent to explore patient views and experiences with antidepressants and thus we selected a group of antidepressant users. This is especially important as antidepressants are known to cause noticeable side-effects [13]. This study therefore aims to explore patients’ experiences and opinions regarding the effect of antidepressants on IBD course as well as attitudes towards future trials with antidepressants.

## Methods

### Setting

South Australia is the fourth largest of Australia's six states and two territories, with >1.6 million inhabitants. The Royal Adelaide Hospital (RAH) is the largest public tertiary teaching hospital in South Australia (SA). At the time of the study, the RAH IBD Service served ~550 IBD patients offering access to IBD nurses and psychologists with an interest in IBD.

### Design and participants

A clinical case-note audit was performed reviewing over 300 of the available IBD patient files as described elsewhere [9]. Inclusion criteria for invitation to participate in this extension of the study were:

1) a diagnosis of IBD (Crohn’s disease (CD) or ulcerative colitis (UC)) by a specialist gastroenterologist

2) listed on the RAH IBD Service database

3) contact with RAH IBD Service within the preceding 6 months and

4) currently taking antidepressants.

Semi-structured in-depth interviews with open-ended questions were conducted.

### Procedure

Of 51 individuals who met the inclusion criteria, a random sample of 15 was selected and invited for interviews. The interviews took place between January and March 2011. The sample size was based on previous literature showing that data saturation is typically reached with groups of 10–15 individuals [14]. In this case, data saturation was reached after the 12th participant was interviewed, however, the researchers decided to conduct the remaining three interviews as they had already been booked. Interviews were performed face-to-face. They were digitally recorded and transcribed verbatim. Transcripts were then checked for accuracy.

### Measurement

A protocol for semi-structured interviews was designed by AM-W in collaboration with the remaining authors. Questions were open-ended and pertained to: the history of IBD and its course, reasons for taking antidepressants and details of this therapy (type, dose, length of treatment, etc.), acceptance of this treatment, patients’ observations in relation to side-effects and impact on IBD (e.g. impact on pain, frequency of bowel movement), observed impact on quality of life (QoL), attitudes towards antidepressants, and attitudes towards future trials with the use of antidepressants. A Visual Analogue Scale (VAS) designed for the purpose of this study was also used as part of the interview (see Additional file 1: Appendix 1) and the data collected as part of it are presented in Table 1 and 2.

**Table 1:** Visual Analogue Scale scores for the acceptability of antidepressants (n = 15) collected as part of the Visual Analogue Scale in 2011

	**Mean (SD)**	**Qualitative score**
**How many side effects do you feel from this drug? (scale 0–100, from none – a lot)**	12.86 (22.05)	Few
**How much do side-effects from this drug bother you? (scale 0–100, from not at all to a lot)**	5.2 (7.4)	Very little
**How much do you like this drug? (scale 0–100, from not at all to a lot)**	60.8 (31.28)	Like it
**Does this drug make you feel more “normal”? (scale 0–100, from definitely no to definitely yes)**	78.57 (24.70)	Yes

**Table 2:** Advantages and disadvantages of the treatment with antidepressants for each patient collected as part of the Visual Analogue Scale in 2011

**Patient No.**	**Advantages**	**Disadvantages**
1	No side effects, efficacy	Nothing
2	Makes me feel happier, stops me getting angry quickly	Nothing
3	Feeling less anxious and depressed especially towards the evening	None
4	Able to sleep better at night	Can make you feel groggy in morning if I haven't slept it off
5	When they do work I feel a lot more confident and secure	If it doesn't work sometimes I break down or become more aggressive
6	Prozac makes me feel that I can cope with day to day activities	Not being able to stop taking Prozac
7	Helps to cope with my illness	Sleep too much
8	Couldn't adequately answer as long time since starting a treatment	Couldn't adequately answer as long time since starting a treatment
9	Stops me running to toilet	Heart burn
10	Less feelings of hopelessness	Stomach pain
11	Relaxation and improvement in sleep	Having to take them
12	I am able to live a better qol with a more positive attitude and energy to partake in day-to-day life activities	Possibility of becoming dependent on it later on in life
13	You do not notice it	Nothing
14	Don't experience the deep depression anymore	Weight gain, don't experience the high enjoyment anymore
15	I don't know I am having the drug	Nothing

### Analysis

A qualitative content analysis [14] was conducted by BS in collaboration with the remaining authors. Firstly, the transcripts were read twice to increase familiarity with the data. Interviewer and respondent dialogue was then transferred into Microsoft Excel (no qualitative methods-specific software was used as Microsoft Excel seemed adequate for the purpose of this analysis), with each question given a separate worksheet, and each uninterrupted segment of dialogue of the interviewer and respondent given a separate cell within the worksheet for a given question. Each segment of dialogue from a respondent was sequentially and systematically coded according to the explicit and implicit response to the given question. Responses were coded in the context of the question, any subsequent prompts or probes used by the interviewer, and the preceding and following segments of dialogue from the interviewee in response to that question. New codes were created when the implicit or explicit content of a given segment of dialogue did not correspond with a previously developed code. Any interrelated questions and their respective codes were allocated to groups based on the similar focus of given sets of questions. Following the coding of all questions in a given group, the codes and general trends were assessed across questions. Any new emerging codes or inconsistencies observed were used to revise the content analysis. Codes were then summarised and quantified where appropriate and the analysis was written up using descriptive statistics and supporting quotes where necessary. Codes were checked by two independent reviewers (BS and AMW), the presence/absence of divergent patient views was discussed and a high inter-rater reliability was achieved. Summary statistics were used to summarise the data from the VAS scale.

## Ethical considerations

This study was approved by the Royal Adelaide Hospital and University of South Australia Research Ethics Committees. Selected patients were invited to the study by their treating doctors and contacted by BS only after they expressed their interest to the treating doctor. Each participant gave written informed consent prior to their interview and patient anonymity was preserved. Patients were given adequate time to familiarise themselves with the information about the study and discuss participation with family and/or friends. They were informed they could withdraw from the study anytime without any consequence for their future treatment at the clinic. The study was conducted in compliance with the Declaration of Helsinki.

## Results

### Patient characteristics

All 15 IBD subjects invited to participate actually participated in the interview study. On average interviews lasted 30 min. Of these, nine (60%) were female. The mean age was 45.8 (*SD = 17.11*) years, with the minimum of 20 years and the maximum of 81 years. Twelve patients (80.0%) had CD, two patients had UC (13.3%), and one patient had colitis of undetermined aetiology. The time since diagnosis ranged from three to 30.5 years (*M* = 16.8, *SD* = 8.9). The number of current symptoms reported per patient ranged from one to seven (*M* = 3.5, *SD* = 2.0). The most commonly reported symptoms were pain (86.7%), diarrhoea (66.7%), nausea (33.3%), fatigue 26.7%, bloating (26.7%), and difficulties tolerating medications (20.0%).

### Antidepressant treatment

#### Reasons for taking antidepressant treatment

Patients reported between one and three (*M* = 1.63, *SD* = 0.81) reasons for taking an antidepressant. The most commonly stated reasons were depression or depressed mood, reported by 10 patients (66.7%), and anxiety or anxious mood, reported by seven patients (46.7%). Other reported reasons included sleeping problems, stress, diarrhoea, anger, Asberger’s syndrome, and obsessive compulsive disorder.

#### Antidepressant treatment history

For the majority of patients (13, 86.7%) their current or recent treatment with antidepressants was the first time they had received the treatment. The duration of antidepressant treatment ranged from four and a half months to 13 years, with an average of 5.6 years (*SD* = 4.8). The most commonly prescribed medications were Amitriptyline and Sertraline, with three patients (20.0%) each, followed by Paroxetine and Escitalopram with two patients (13.3%) each, and Duloxetine, Nortriptyline, Fluoxetine, Citalopram, and Mirtazapine being used by one patient each.

The majority of patients (10, 66.7%) reported experiencing no side effects from antidepressant therapy. Five patients (33.3%) reported experiencing between one and three distinct side effects (*M* = 2.3, *SD* = 0.6), with two patients reporting dry mouth, and one patient each reporting diarrhoea, abdominal pain, headaches, weight gain, heart burn, emotional numbness, and anhedonia. One patient reported a cessation of the side effects of antidepressant treatment after a change in the type of antidepressant he was using from Mirtazapine to Sertraline. Three patients (20.0%) discussed the difficulty in dissociating symptoms that may be side effects of antidepressant treatment from the side effects of other medications they were using, or IBD symptoms.

“It can be difficult to tell when you’ve got something like Crohn’s because anything could be an effect of the- I’m on three different medications. Four if you count the Loperamide. So anything could be a side effect of one of those or it could be the Crohn’s itself.” (Patient 14)

#### The efficacy of antidepressant treatment

When asked if treatment with antidepressants had helped them or improved their QoL, all fifteen patients indicated that it had. The areas of improvement cited were primarily psychological, but some patients indicated social as well as biological areas of improvement in their lives. Three patients expressed some uncertainty in dissociating the causes for their improvements from the three areas of antidepressant treatment, other psychotropic medications, and psychological treatment. The most common area of improvement was a reduction in anxiety, anxious mood, or stress, and an increase in the ability to relax, reported by ten patients (66.7%).

“…it definitely curbs… you know the anxiety sort of panic attack type stuff… Look I’d have to say it’s working cause I don’t notice it anymore so…” (Patient 5)

Eight patients (53.3%) reported a reduction in depression or depressed mood.

“I mean I tend to get fairly depressed in the evenings. More than anything, you know, it does tend to help that…” (Patient 6)

Five patients (33.3%) felt that antidepressant treatment had reduced their irritability or anger.

“Definitely stops me from being bad-tempered or moody.” (Patient 2)

Four patients (26.7%) described subsequent improvements in: their capacity to do more housework, paid work, or education; their ability to engage with partners, family, or friends; and their capability to participate in social or recreational activities.

“…I think my relationship with my husband has improved a lot because I… when I’m at my lowest, I’m pushing him away. Whereas, now that I’m actually… feeling closer towards him and, you know, feeling like I want him to be part of my life now more than I did at the beginning of the year when I wanted to push him away so… I think it improves relationships. I mean I guess that’s just because of my mental attitude has changed so much.” (Patient 1)

Similarly, two patients reported increased energy and motivation to engage in these activities.

“So I definitely feel more energy. I’m positive now. I don’t look at everything as a negative anymore. If I have a flare-up, I might get down about it. But then, you know, move on straight away. I’m still doing things like working, making an effort to go to my shifts even when I’m unwell. I’m just pushing myself that little bit extra. So it’s definitely given me more of a quality of life.” (Patient 4)

Three patients (20.0%) felt that the antidepressant treatment had improved their cognitive processes by giving them increased control over their thoughts, improving the clarity of their thoughts, and reducing irrational thought processes. Three other patients described improvements in their sleeping patterns, although this may have been associated with coinciding improvements in other areas of functioning such as improvements in the ability to relax and reductions in the experience of anxiety and pain.

“Yep. I find I relax a lot better at night. I get a better night’s sleep. There’s obviously still nights where I toss and turn and I wake up and the ileostomy could be playing up, so that’s a big factor in the sleep problem too. But otherwise, nah I feel like I’ve got some sort of routine within my body so I do get at least seven hours of sleep at night instead of the two.” (Patient 12)

Finally, three patients also discussed how the antidepressant treatment had helped them cope with the stressors in their life, particularly those posed by their disease.

“Yes… It’s helping me cope with what’s wrong with me. It’s hard to- to deal with sometimes…” (Patient 9)

#### Improvements in disease function

Three patients (20.0%) raised, unprompted, the feeling that the antidepressant treatment had ameliorated physical symptomatology associated with their disease, including reducing the frequency of symptoms or flare-ups of disease.

“And I honestly believe it helps me with this disease.” (Patient 2)

When asked specifically whether the antidepressant had influenced their IBD in terms of pain, frequency of bowel movement, or frequency of relapses, 10 patients (66.7%) felt that the antidepressant treatment had not influenced their disease course. Two patients (13.3%) described again how it was difficult to dissociate the effects of the antidepressant on the disease course from other possible causal mechanisms. Of the five patients who felt the antidepressant had influenced their disease course, three discussed how the reduction in feelings of stress mediated the influence of the antidepressant on improving the disease course.

“… when the OCD was full-blown, naturally that was incredibly stressful and I was very sick at the time. So I suppose the main effect of it is just to calm me down and take away that aspect.” (Patient 14)

Of the five patients (33.3%) who felt the antidepressant had improved their disease course, three patients described a reduction in pain, two described reductions in the frequency of bowel movement, one described a reduction in nausea, and one patient also felt that the frequency of relapses of disease had reduced.

#### Attitudes towards antidepressants

Patients’ responses on the VAS summarising their acceptance of antidepressants are presented in Table 1. Patients reported a reasonable level of acceptance of the treatment with antidepressants. Patients’ perceived advantages and disadvantages of the treatment are listed in Table 2.

Despite every patient indicating earlier that antidepressants had increased their quality of life, patients’ views or attitudes towards antidepressants in general were not as unilaterally positive. Nine patients (60.0%) had a generally positive attitude, four patients (26.7%) were ambivalent, and two patients (13.3%) held a negative view towards antidepressants. Nine patients (60.0%) felt that the potential benefits need to be contrasted against the costs of antidepressant treatment in the individual context of each person. Potential benefits listed included an improvement in mood and functioning, while the potential costs included side effects and the concern of becoming dependent. Some patients also raised the concern that while antidepressants may superficially fix a problem, they did not solve the underlying cause.

“Obviously because they can be addictive and, from what I hear, you know you get on them and then you think you’re okay so you stop them and then you go downhill again and you gotta basically stay on them to flatline. But, yes I didn’t want them. I don’t wanna be walking around zombified or making some pill make me feel happy when it shouldn’t be a pill. It should be just because life’s good… It just depends on the person I suppose.” (Patient 12)

One patient felt that there may be a perception that antidepressants are a ‘magic pill’ which may be perpetuated by doctors and could need dispelling.

“Yeah. Cause it’s like “Okay take this pill and-” Not that they say that but… you know, it’s more or less “Alright take this medication and things are going to get better” and stuff like that, so you have your hopes up high and if you don’t have a GP that’s on it and knows what they’re doing and what ones are best for you… like I think you can get worse off than where you first started. But I’m sure they are beneficial, I mean I know people that have been on them… that have had a lot of benefit out of them… I just wasn’t one of those people I guess.” (Patient 3)

Despite the sizeable minority who expressed ambivalent or negative attitudes regarding antidepressant use, fourteen patients (93.3%) stated that they would recommend antidepressants to other IBD patients. However, of these, four patients (36.4%) expressed some reluctance and placed certain provisos on this recommendation, whereby antidepressant treatment would depend on the individual’s circumstances.

#### Potential clinical trials

Finally, patients were asked whether they would be willing to potentially participate in a clinical trial if there were evidence for antidepressants to play a role in the management of IBD. Twelve patients (80.0%) stated that they would while three would not. Six patients (40.0%) stated that they would in order to help improve treatment for other IBD patients. Four patients (26.7%) said they would be interested in order to improve their own quality of life.

Of the factors which detracted from their willingness to participate in a future trial, two patients felt that the side-effects of antidepressants were not worth the potential benefit.

“…for some reason medication doesn’t sit well with me. And like I’ve got to the point now where, as I said, I’d rather just live with my disease than all the other side effects that come from all the… medication.” (Patient 3)

Two patients did not want to change their current antidepressant medication due to their success with it.

“I might but as I say I’m so suited with the Zoloft I’m sort of edgy about the idea of changing it.” (Patient 14)

One patient (6.7%) felt that because their antidepressant use hadn’t affected their IBD in any way they would not wish to participate, and one patient felt too unwell to undertake the travel commitment necessary for participation in a trial.

## Discussion

This paper is the first to explore patients’ views on the role of antidepressant drug therapy in IBD and one of very few human clinical studies exploring the use of antidepressants in IBD sufferers.

The paper documents that IBD patients are prescribed antidepressants for mental health issues rather than somatic symptoms which contrasts slightly with their typical use in patients with functional gastrointestinal disorders (FGIDs), and irritable bowel syndrome (IBS) in particular, who are frequently offered antidepressants to treat both somatic and psychological complains [15]. Due to the proposed aetiology of IBS symptoms which includes psychological factors and also thanks to several studies into the efficacy of antidepressants in FGIDs, antidepressants have now become a part of the standard treatment for IBS, offering significant benefits to patients [16]. Previous work has shown that gastroenterologists commonly use antidepressants in IBD patients for symptoms commonly encountered in IBS [8]. IBD and IBS share symptomatology and some other characteristics [17] and some researchers have argued they may, in fact, be two different ends of the spectrum of the same condition; one with lesser symptoms and greater inflammation, the other with greater symptoms and minimal inflammation [18,19].

Moreover there is now a growing appreciation that IBS can occur in healed IBD [11,12] and that without endoscopic examination, the precise cause of symptoms in a patient with IBD is frequently uncertain at any particular point in time. Yet, there are very few studies that have critically examined the role of antidepressants in IBD. In support of the concept that antidepressant therapy may be of real efficacy in IBD, and in fact offer a more specific benefit here than in IBS, several studies have now reported anti-inflammatory properties in certain antidepressants [5,20,21]. Moreover, in animal models antidepressant therapy has now been documented to ameliorate visible GI inflammation [22,23]. Thus, antidepressants may potentially offer additional benefit to IBD patients with active inflammation, assisting with mental health but also directly reducing inflammation. For this reason clinical trials in this area are needed and are likely to be conducted in the future.

However, since antidepressants have been shown to have a high occurrence of side-effects [24,25], many leading to medication changes or intolerance [26], it seems prudent to examine patients’ perspectives on the use of antidepressants in IBD and collect their observations prior to planning future trials, to better inform their design. This paper has shown that patients do not report many side-effects and if they experience any, these do not seem to significantly impact them. However, one of the weaknesses of the current study is that we have, as yet, only sought the opinions of patients who have chosen to continue to take antidepressants (leading perhaps to an overly optimistic outlook). In future, it would also be informative to seek to interview those who either were not offered antidepressants, and others who may have been prescribed these agents, yet did not continue therapy. Patients also report medication side effects from standard IBD treatments and it is sometimes difficult to judge which side-effect comes from which medication. Clearly, this is a highly medicated group of patients and thus the risk of causing more side-effects must be considered when planning future studies. Yet, this group of interviewed patients lists more advantages than disadvantages of treatment with antidepressants.

Even though patients observe benefits to their mental health and overall quality of life, only five of them commented on the drug impacting on their disease course. In the remaining patients, the medication was thought to offer psychological benefit, also with respect to motivation and cognitive functions. In terms of physical symptoms, antidepressants were noted to improve sleep. This observation was previously made by interviewed gastroenterologists [8] who emphasised that their patients while on antidepressants had not reported needing to visit a toilet during night with better controlled bowel functions – which could be either due to better sleep or improved disease control or both – emphasizing the difficulty in present data in determining whether antidepressants improve only symptoms, or also disease activity. The five patients reporting reduction in IBD symptoms, and improvement in pain, fewer bowel movements and less frequent relapses of IBD in particular, interestingly conceptualised that this was due to a reduction in perceived stress. And this was thought to mediate the influence of antidepressants on disease course. This observation will need to be however confirmed in clinical trials as the present design does not allow for testing this hypothesis.

With respect to patients’ attitudes towards antidepressants, these were largely positive. However, patients discussed their worries that the treatment could be only superficial, offering relief while ongoing and they clearly feared dependence. There is an ongoing debate on whether antidepressants may in fact cause dependence [27], and in any case, presenting problems when tapering off [28], with significant numbers of patients no longer needing an antidepressant for their mental health problem yet suffering unbearable withdrawal effects while discontinuing and thus remaining on treatment [29]. Nevertheless, patients participating in this study reported antidepressants to be a medication worth recommending to fellow IBD sufferers as long as the decision of their use was taken after consideration. Although studies exploring attitudes to antidepressant use in larger samples or in samples recruited in primary care (and thus with possibly better controlled IBD) are not available, studies conducted in the general population in primary care showed a less receptive attitude to antidepressants. For example, a survey of 1,054 primary care users showed that over 20% of them did not disclose depressive symptoms to their doctors out of the fear antidepressants will be prescribed [30]. Other studies have reported non-adherence to treatment with antidepressants due to patient beliefs or misconceptions about this type of medication [31,32]. In light of these findings, the positive attitudes to antidepressants identified in the present study should be interpreted with caution and confirmed by larger quantitative studies with more representative IBD samples.

The most positive outcome in this study was patients’ positive attitude towards clinical trials with the use of antidepressant. Overall, 80% of this study’s participants reported willingness to participate in such trials with the hope such studies could help other people but also their own quality of life. Those few rejecting the idea of participating in such a study reported lack of faith in antidepressants really being effective in IBD or claiming the benefits were not worth the harm caused by the side-effects. However, these patients agreed to participate in the present research study and thus there is a potential that they are generally more willing to participate in studies than an average IBD patient and thus this result should be interpreted with caution. Further studies should be conducted to explore the attitudes of general IBD population towards antidepressants.

## Conclusion

Antidepressants seem to be well tolerated by IBD patients. One third of patients perceived an improvement of their IBD under the influence of this treatment. The positive attitude towards antidepressants in these participants may make the conduct of clinical trials to further assess for any specific role on IBD course feasible. However, due to a small sample size, a qualitative nature of this study and in light of the results of studies on other populations indicating reluctance to taking antidepressants at least in some patients, these results should be interpreted with caution until confirmed in quantitative studies.

## Competing interests

Authors declare that they have no competing interests in relation to this study.

## Authors’ contributions

AAMW contributed to the design of the study, data analysis and interpretation, prepared the first and approved the final draft. ALG contributed to the design of the study, data interpretation, provided feedback on drafts and approved the final draft. BJS contributed to the design of the study, data collection, analysis and interpretation, provided feedback on drafts and approved the final draft. JMA contributed to the design of the study, data interpretation, provided feedback on drafts and approved the final draft. All authors read and approved the final manuscript.

## Authors’ information

AAMW is a research psychologist working in the area of Psycho-gastroenterology with a particular interest in testing psychosocial interventions to manage IBD and other chronic gastrointestinal conditions. She is the author of two systematic reviews and other research papers on the role of antidepressants in IBD. ALG is a pharmacologist with an interest in substance use and mental health and has an expertise in pharmacology of antidepressants. BJS is a psychology doctoral student with an interest in psychotherapeutic and psychiatric interventions to manage chronic gastrointestinal and hepatologic conditions. JMA is an Associate Professor of Medicine and a Senior Clinician in Gastroenterology, Head of IBD Service with an interest in biopsychosocial therapies to co-manage IBD and other gastrointestinal conditions.

## Pre-publication history

The pre-publication history for this paper can be accessed here:

http://www.biomedcentral.com/1471-230X/12/93/prepub

## Supplementary Material

Additional file 1: APPENDIX Visual analogue scale regarding the use of antidepressants. Click here for file
