# Supplementary material for: A magic pill? A qualitative analysis of patients’ views on the role of antidepressant therapy in inflammatory bowel disease (IBD)
Source: BMC Gastroenterol. 2012 Jul 20;12:93. doi: 10.1186/1471-230X-12-93 (PMC3444888; doi:10.1186/1471-230X-12-93)
Supplement: Additional file 1: APPENDIX — Visual analogue scale regarding the use of antidepressants. [file 1471-230X-12-93-S1.doc]

**APPENDIX - VISUAL ANALOGUE SCALE REGARDING THE USE OF ANTIDEPRESSANTS**

All questions in this questionnaire are about what has happened to you since you started antidepresssnats. Any information you give here is completely confidential. Please answer all questions honestly and accurately.

##### Please make a vertical line across each line below to show what you think about

Office Use Only

##### the antidepressants you are taking.

A1. How many side effects do you feel from this drug?


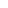


none a lot

A2. How much do side effects from this drug bother you?


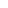


not at all a lot

A3. How much do you like this drug?


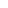


not at all a lot

A4. Does this drug make you feel more “normal”?


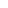


definitely no definitely yes

A5. What are the best things about this drug?

____________________________________________________________________________

____________________________________________________________________________

A6. What are the worst things about this drug?

____________________________________________________________________________

____________________________________________________________________________

Investigator Signature: ………………………….. Date: ……………………………
